# Technologies for profiling the impact of genomic variants on transcription factor binding

**DOI:** 10.1515/medgen-2021-2073

**Published:** 2021-08-14

**Authors:** Janna Leiz, Maria Rutkiewicz, Carmen Birchmeier, Udo Heinemann, Kai M. Schmidt-Ott

**Affiliations:** Charité–Universitätsmedizin Berlin, Corporate Member of Freie Universität Berlin and Humboldt-Universität zu Berlin, Department of Nephrology and Intensive Care Medicine, Hindenburgdamm 30, 12203 Berlin, Germany; Max-Delbrück-Center for Molecular Medicine in the Helmholtz Association (MDC), Molecular and Translational Kidney Research, Robert-Rössle-Str. 10, 13125 Berlin, Germany; Max-Delbrück-Center for Molecular Medicine in the Helmholtz Association (MDC), Macromolecular Structure and Interaction, Berlin, Germany; Max-Delbrück-Center for Molecular Medicine in the Helmholtz Association (MDC), Developmental Biology and Signal Transduction, Berlin, Germany

**Keywords:** transcriptional regulation, genomic variants, TF:DNA binding, binding prediction

## Abstract

Transcription factors (TFs) bind DNA in a sequence-specific manner and thereby regulate target gene expression. TF binding and its regulatory activity is highly context dependent, and is not only determined by specific cell types or differentiation stages but also relies on other regulatory mechanisms, such as DNA and chromatin modifications. Interactions between TFs and their DNA binding sites are critical mediators of phenotypic variation and play important roles in the onset of disease. A continuously growing number of studies therefore attempts to elucidate TF:DNA interactions to gain knowledge about regulatory mechanisms and disease-causing variants. Here we summarize how TF-binding characteristics and the impact of variants can be investigated, how bioinformatic tools can be used to analyze and predict TF:DNA binding, and what additional information can be obtained from the TF protein structure.

## Introduction

Transcription factors (TFs) are regulatory proteins that bind DNA in a sequence-specific manner. Along with chromatin accessibility and histone modification, TFs regulate the expression of target genes depending on cell type, developmental stage, or external signals; for a detailed review on the regulatory epigenome and histone modifications see [1], [2].

Over 1,000 potential TFs have been identified in humans. In general, TFs are highly conserved among species and recognize specific nucleotide sequences or motifs in non-coding regulatory regions of the genome. Binding is based on the complementarity of the DNA sequence and protein structure. As recognition sites are rather short, usually 6–12 bases, and every TF has numerous binding sites throughout the genome, many TFs bind cooperatively as multimers to ensure highly specific and stable interactions. To add an additional layer of control to the rigorously regulated process of gene expression, many TFs recruit cofactors or depend on binding of specific ligands [3].

**Figure 1:**
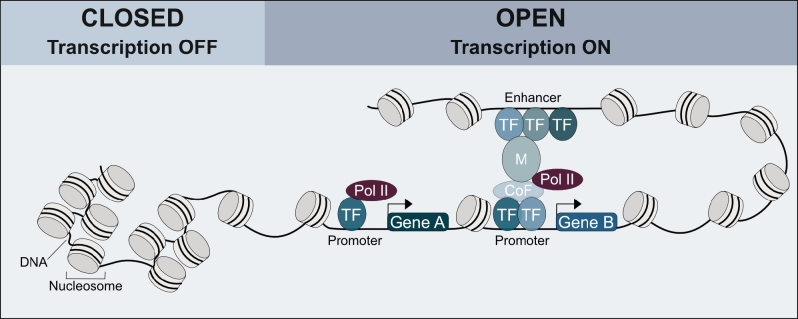
Transcriptional activation is mediated by chromatin state and transcription factor binding. Densely condensed chromatin (closed) prevents transcription factors and other proteins needed to initiate transcription from binding, thereby inhibiting gene expression. Open chromatin on the other hand is accessible for proteins to bind to promoter and enhancer regions. Transcription factors can either directly guide RNA polymerase II to promoter regions of target genes or work in cooperation with other factors and mediators to assemble a transcription initiation complex. TF, transcription factor; Pol II, RNA polymerase II; CoF, cofactor; M, mediator.

Though the majority of the genomic DNA is usually densely packed in nucleosomes and higher-order structures, making it inaccessible to TFs, a special class of TFs, called “pioneer factors,” are able to bind to their recognition sites even in condensed chromatin. They thereby induce changes to the chromatin structure that enable binding of other factors needed to initiate transcription [4]. To amplify the expression of a protein-coding target gene, TFs guide RNA polymerase II to gene promoter regions to start the transcription process (Figure 1). TFs can also block binding sites for other proteins and thereby, depending on the specific context, act as repressors. How DNA sequence variants in TF binding sites impact site recognition and transcriptional regulation is difficult to predict.

Though we tend to think of TFs as either occupying particular sequences in the chromatin or not, many TFs are controlled in a more complex manner. Some TFs are produced in response to external or internal stimuli in a pulsed, oscillating, or sustained manner. Consequently, the genes that are switched on by such TFs depend on their expression dynamics [5], [6]. The TF MyoD, a master regulatory factor of skeletal muscle known for its ability to initiate the muscle-specific differentiation program, is one example. In proliferating muscle precursor cells, MyoD oscillates with a periodicity of 2–3 hours, thereby keeping the cells in an undifferentiated state. This oscillatory MyoD pattern changes before the cells differentiate. When MyoD expression becomes sustained, cells start to differentiate and undergo fusion into myotubes [7]. This implies that the biological function of TF binding should not only be investigated statically, but in a time resolved fashion.

The impact of mutations in genes coding for TFs and resulting structural changes of the proteins have been investigated and linked to diseases in many studies. Because the protein-coding exome covers less than 2 % of the human genome, the focus of variant analysis has been expanded to non-coding regulatory regions (Krude et al. this edition, Guo et al. this edition). Approximately 80 % of all genome-wide association study (GWAS) hits affect the non-coding DNA and many of them are thought to act through differential TF binding [8]. Although it is often challenging to identify disease-associated variants and to prove causation, several studies show that variants in the regulatory genome alter TF:DNA interactions and are associated with altered target gene expression and disease [9], [10]. This emphasizes the necessity of examining genetic variants in the context of patient clinical phenotypes. For example, single-nucleotide variants (SNVs) in the promoter region of coagulation factor F9 have been associated with a specific subtype of the blood-clotting disorder hemophilia B. More than 20 different SNVs in three distinct clusters affecting highly conserved base pair positions in the binding sites of the TFs HNF4α, ONECUT1/2, and C/EBPα have been identified. All three binding sites are in close proximity, and disruption of either one of them significantly decreases promoter activity, indicating a possible cooperation of HNF4α, ONECUT1/2, and C/EBPα to control F9 expression. This example shows how alterations of single bases in TF-binding motifs can disrupt TF:DNA interactions and critically alter gene expression leading to disease [11].

Here, we review different *in vitro* and *in vivo* techniques that can be used to investigate TF:DNA binding characteristics and describe how the impact of variants on the binding capacity can be measured. We outline the recently made advances of bioinformatic tools and machine learning approaches to predict and analyze TF-binding sites (TFBSs) in the human genome. We further review techniques for deriving quantitative TF:DNA affinity data and discuss how they can be related to high-resolution structures of TFs bound to their DNA targets. We finish by discussing future challenges relating structures of target DNA-bound TFs with quantitative affinity data.

## How can transcription factors and their binding motifs be investigated?

There are several techniques to identify potential regulatory domains with TFBSs and to assay chromatin accessibility on a genomic scale. Widely used in an ever-increasing number of publications are chromatin immuno-precipitation followed by sequencing (ChIP-seq) and the assay for transposase-accessible chromatin using sequencing (ATAC-seq) (Guo et al. this edition).

ChIP-seq enables the study of protein:DNA interactions and generates genome-wide maps of TFBSs or histone modifications. The technique depends on cross-linking a protein of interest, e. g., a specific TF, to DNA which is then fragmented. Using specific antibodies targeting the protein of interest, protein-bound DNA fragments are isolated and can be sequenced and mapped back to the genome. ChIP-exo, a modification of the ChIP-seq protocol, uses an exonuclease to trim the TF-bound DNA, thereby improving the signal-to-noise ratio and the resolution of TFBSs [12]. ChIP experiments can be utilized for *de novo* motif discovery and provide information about sites of high transcriptional activity and potential TF target genes in the cell of interest [13]. Data are already available for a multitude of TFs in many different cell types, tissues, and conditions. Information about TF:DNA interactions and discovered motifs are stored in numerous open-access databases, such as JASPAR [14], and can be employed for genome-wide studies (Garda et al. this edition).

ATAC-seq was first established in 2013 and provides a robust and uncomplicated alternative for methods such as DNase I hypersensitive sites sequencing (DNase-seq) and formaldehyde-assisted isolation of regulatory elements coupled with sequencing (FAIRE-seq), which require time-consuming sample preparations and high amounts of input material. ATAC-seq uses a hyperactive mutant of the prokaryotic Tn5 transposase to cleave DNA in open chromatin regions and ligate it to sequencing adaptors. Labeled DNA fragments are then purified, amplified, and sequenced, providing genome-wide profiles of chromatin accessibility [15] (Guo et al. this edition).

Recently, ATAC-seq has been used in single-cell approaches to further broaden the scope of experiments and data resolution. In combination with single-cell RNA sequencing, this is a powerful tool to combine epigenomics with transcriptomics and study cellular heterogeneity of the regulatory landscape [16].

## How can we measure transcription factor:DNA interactions and the impact of DNA variants on transcription factor binding?

The ChIP-seq techniques map the TF-binding DNA sites in a genome-wide fashion, but result in low spatial resolution and permit only limited assessment of binding strength. They can also show some unspecific and/or non-functional binding. That is why *in vitro* techniques are employed to accurately measure TF:DNA interactions and the impact of DNA variants on TF-binding. Ideally, *in vitro* assays used for this purpose are able to accurately determine the dissociation constants for both high- and low-affinity binding [17]. A further issue is the scalability of the assay. Screening large numbers of variants is still a challenge in terms of cost and time efficiency.

Such tests require homogeneous and purified protein samples. As it is often difficult to express full-length TFs at high levels [18], only the DNA-binding domain (DBD) is usually expressed in a host system and further purified using high-performance chromatography. To facilitate the purification, a peptide tag, most commonly hexahistidine (His6) or glutathione *S*-transferase (GST), is fused to either the N- or the C-terminus of the protein. In some cases, creating a fusion protein that includes an additional solubility tag is necessary, and commonly the purification and solubility tags are proteolytically removed before the final purification stage, as they may interfere with DNA binding or otherwise alter protein properties.

**Figure 2:**
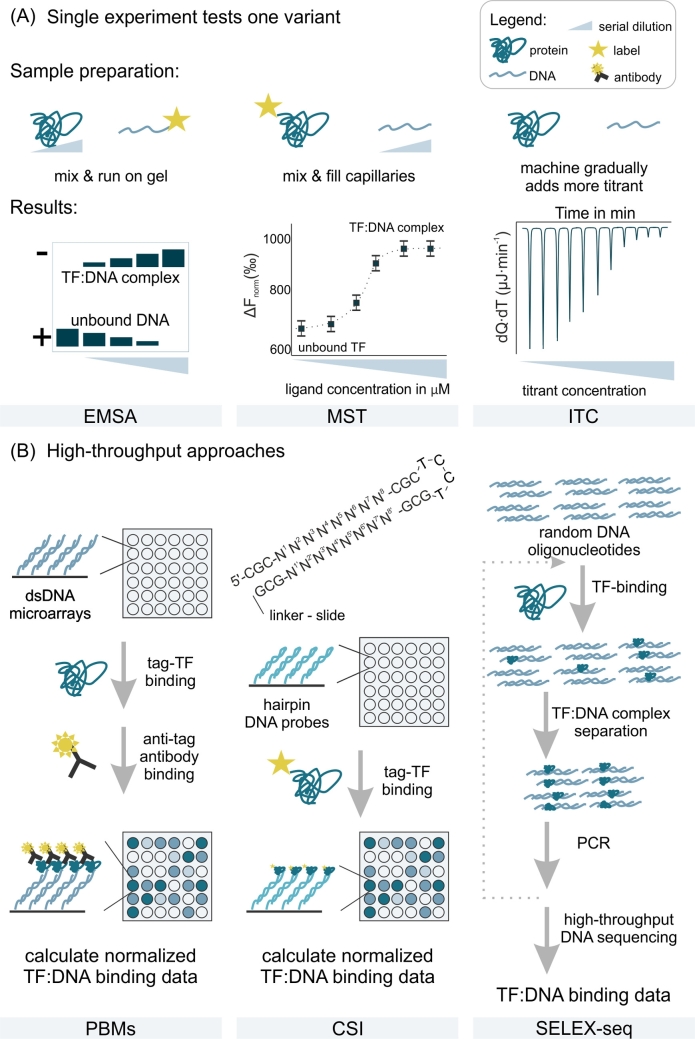
Selection of methods used to experimentally assess TF:DNA interactions. **(A)** Simplified functional scheme and an example of typical results from methods for investigating specific DNA sequences: electrophoretic mobility shift assay (EMSA), microscale thermophoresis (MST), and isothermal titration calorimetry (ITC). **(B)** Simplified schemes of high-throughput approaches such as protein-binding microarrays (PBMs), Cognate Site Identifier (CSI), and evolution of ligands by exponential enrichment coupled with sequencing (SELEX-seq).

The electrophoretic mobility shift assay (EMSA) is among the basic assays used for *in vitro* studies of protein binding to DNA. EMSA is a simple laboratory method where the change in migration between free and protein-bound DNA through a polyacrylamide or agarose gel is assessed, as the speed of migration depends on the size, charge, and, to a lesser extent, the shape of the analyzed molecules or complexes. However, EMSAs are not suitable for analysis of large numbers of DNA variants and do not easily yield dissociation constants. Fluorescence anisotropy binding assays, on the other hand, enable the determination of quantitative binding affinity data, but they require the DNA variants to be fluorescently labeled, which makes them suboptimal for analyzing a large number of DNA variants (Figure 2A).

Methods that have been successfully used to determine dissociation constants (K_D_ values) for TF:DNA affinity include isothermal titration calorimetry (ITC) and microscale thermophoresis (MST). Due to the sensitivity of K_D_ measurement ranging from pM to mM, MST was proven to be effective in quantitating both high- and low-affinity binding events. It usually requires fluorescently labeled protein, which may be easily obtained as a fusion protein, e. g., GFP-TF. For the ITC experiment neither the TF nor the DNA needs to carry a fluorescent label. Nonetheless, larger amounts of both TF and DNA variants are required to determine the affinity (Figure 2A). However effective, neither ITC nor MST can be considered as optimal for testing a large number of variants in a single experiment as it only determines the affinity of one ligand.

To test tens of thousands of double-stranded DNA (dsDNA) molecules, protein-binding microarrays (PBMs) are most often used. Their main limitations are a lack of commercially available chips and the need for a rather stringent washing step, which may result in loss of TFs binding with low affinity (Figure 2B). An interesting variation of PBMs is Cognate Site Identifier (CSI), which is based on preparing a microfabricated array displaying every permutation of a dsDNA sequence of up to ten positional variants, assessing the effect on binding of each possible variant in the same experiment. For more detailed information about other methods that measure binding events occurring on a surface see references [19], [20]. Those methods are proven to be effective for all ranges of TF:DNA binding affinities, but it should be noted that they often require usage of complementary methods that determine affinity in solution [17].

Another approach to high-throughput measurements of TF:DNA interactions is based on the generation of high-complexity libraries of random or genomic DNA fragments. Systematic evolution of ligands by exponential enrichment (SELEX) is commonly used for development of the library (Figure 2B). It starts with the synthesis of a gigantic library of randomly generated oligonucleotides flanked by constant 5′ and 3′ ends that serve as primers. After addition of the TF of interest, the sequences that did not bind are removed by affinity chromatography or by capturing the target on paramagnetic beads. The bound sequences are amplified by PCR and can undergo another round of more stringent selection or can be sequenced immediately, depending on the method variant. To learn more about library-based high-throughput TF:DNA interaction measurement methods see reference [21].

## How can bioinformatics approaches predict transcription factor binding sites and the impact of non-coding variants on binding?

Bioinformatics approaches facilitate processing of large datasets and data integration to exploit the potential of a growing body of data sources in order to predict TFBSs and the impact of human genomic variants. The input to such bioinformatic tools includes high-throughput data from methods that map DNA binding specificities of TFs. The most widely used datasets for this purpose are derived from ChIP-seq, ChIP-exo, or SELEX experiments. Potentials for the integration of such datasets with additional databases are abundant (Garda et al. this issue). For instance, they can be combined with data that more broadly map open chromatin based on DNA footprints, such as from ATAC-seq or DNase-seq. In addition, the information can be overlaid with databases that cover disease-associated variants with the goal of identifying potential disease mechanisms (Garda et al. this edition).

**Figure 3:**
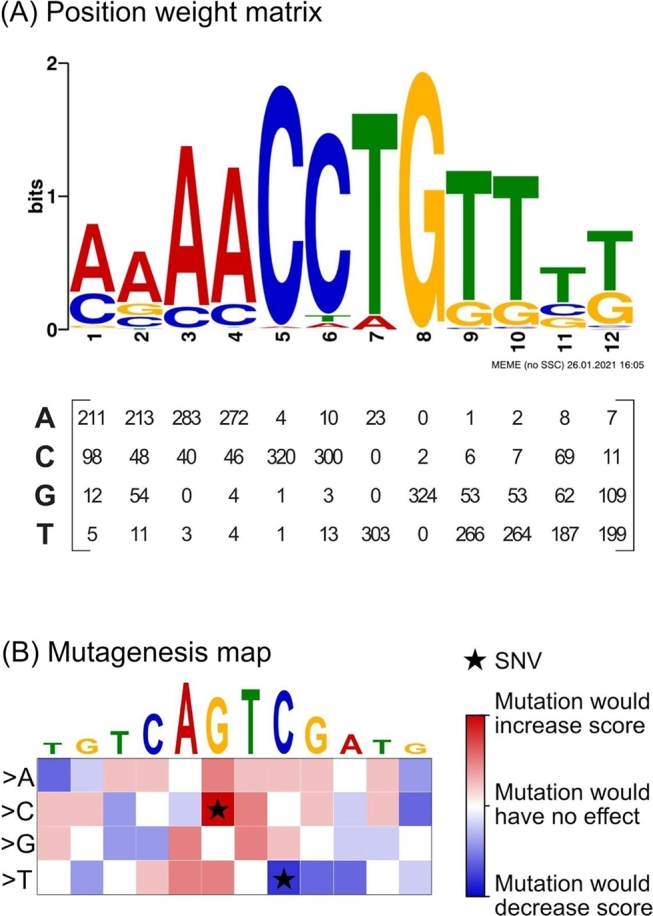
Position weight matrices and mutation maps provide information about transcription factor-binding motifs and the impact of single-nucleotide variants. **(A)** Position weight matrix of a transcription factor derived from *de novo* motif discovery provided by MEME. **(B)** Example of a mutation map highlighting the predicted impact of sequence variants in a heatmap (red indicates increased binding; blue indicates decreased binding). Single-nucleotide variants are highlighted in the map. SNV, single-nucleotide variant.

To discover or map TFBSs and predict the impact of non-coding variants on binding, a multitude of bioinformatic methods is available. For example, enumeration-based approaches (e. g., Yeast Motif Finder) map word-like definitions of motifs across chromatin sequences. They may introduce degenerate positions and motifs of variable length, but they are computationally intense and lack flexibility [22]. In contrast, probabilistic methods work with positional weight matrices (PWMs), which assign probabilities to each base of the DNA sequence at different positions. Analysis of large sets of TF-associated DNA sequences can be used to *de novo* predict PWMs associated with this TF. This can be achieved by PWM-based tools, such as MEME (Figure 3A) [23]. These approaches are limited in the setting of very large datasets and highly depend on the TF under scrutiny [24].

Recent years have witnessed dramatic advances in the field of deep learning, which offers scalable and flexible computational approaches for pattern discovery and operates on large amounts of sequence data. Deep learning enables integration of high-throughput datasets of TF-binding and open chromatin with databases of genomic variants to facilitate prediction of DNA–protein associations and to anticipate the impact of non-coding variants on these interactions. For instance, DeepBind uses convolutional neural networks to predict DNA–protein interactions [25]. In this approach, which is based on tools originally developed for image classification, genomic sequences are treated as fixed-length sequence windows composed of four channels (A, C, G, T). DeepBind uses a set of sequences of variable lengths and, for each sequence, an experimentally determined binding score, which can be quantitative measurements or binary class labels. For training, DeepBind is supplied with large DNA sequence datasets, often of terabyte size, which can be derived from diverse approaches, including ChIP-seq and SELEX. Following training, DeepBind can score new sequences, including human genome regions from reference genomes or from individuals with genetic diseases. Recently, improved deep learning-based approaches have been developed to provide single-nucleotide resolution maps, e. g., a mutation map (Figure 3B). They consist of an importance score for each nucleotide variant at each position that is directly linked to prediction and therefore provide an easy-to-interpret visualization of the predicted variant effect on the binding affinity (for a recent review see [26]).

**Figure 4:**
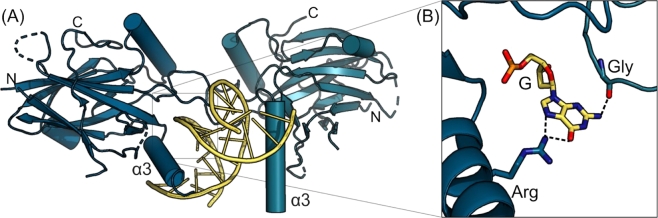
Crystal structure of GRHL1 (PDB ID: 5MPF) in complex with a DNA fragment. **(A)** Binding of the Grainyhead Like Transcription Factor 1 (GRHL1) dimer (shades of blue) to its DNA target site and the shape complementarity between TF and DNA (yellow). **(B)** Example of hydrogen bonds crucial for target sequence recognition.

## Structural studies of binding site recognition by transcription factors: How do they relate to quantitative affinity data and how can they inform binding site prediction algorithms?

High-resolution structures of target DNA-bound TFs yield tremendous insight into the molecular basis of these proteins’ ability to recognize their specific promoter or enhancer sequences and to discriminate against non-cognate sequences or close variants, e. g., SNVs, of the target sequence. Determination of these structures relies on sophisticated, well-established, but low-throughput analytical techniques: X-ray diffraction yields the highest spatial resolution, but requires single crystals of the TF:DNA complex; nuclear magnetic resonance (NMR) spectroscopy provides structures with more limited spatial resolution, but also yields insight into complex dynamics; and single-particle cryo-electron microscopy (cryo-EM) has recently joined the methods allowing to derive atomic models of macromolecular complexes [27], [28]. Although a large number of TF:DNA complex structures are known and deposited in the Protein Data Bank (PDB),1https://www.rcsb.org/ for all the structural detail they reveal these structures are of limited use for assessing the impact of SNVs on TF binding because they typically only display TF binding to high-affinity and consensus DNA sites, leaving non-specific binding or SNVs unstudied.

Crystal, NMR, or cryo-EM structures of TF:DNA complexes allow an intimate view into the protein:DNA interface that determines binding specificity (Figure 4A). The most obvious source of specificity is the pattern of hydrogen bonds formed by the protein backbone or side chains with the polar base pair edges exposed in the grooves of dsDNA. Hydrogen bonds and salt bridges to the sugar-phosphate backbone of the DNA are generally non-specific, but contribute to the overall TF affinity for its target site (Figure 4B). Van der Waals contacts between TF and cognate DNA are less directed; collectively, they define the shape complementarity between TF and dsDNA and thereby contribute to the overall binding energy.

## Future challenges

A particular challenge rests in relating structures of target DNA-bound TFs with quantitative affinity data as described above. It is usually possible to ascertain that the structure of a TF:TFBS complex is biologically relevant by mutating key residues of the protein or the DNA as identified by structure analysis and assaying the subsequent binding behavior. Taking the example of the Grainyhead/CP2 transcription factors, proteins of core interest to us, both protein and DNA variants lacking crucial interacting residues were clearly deficient in binding as determined by EMSA, ITC, and *in vivo* reporter assays [29]. However, it is considerably more difficult to derive accurate binding energies for a TF:DNA complex from crystal structures. The main reason for this lies in the fact that the various contacts at the TF:DNA interface are closely related to the thermodynamic binding enthalpy, but do not allow to assess the entropic contributions to the free energy of binding, which arise from restricting the conformational space of TF and DNA in a complex and from solvation and desolvation effects, and hence the dissociation constant of a complex.

The ability to derive affinity parameters from TF:DNA structures is crucial for supporting efforts towards predicting the consequences of SNVs for gene regulation through a specific TF. If these predictions aim to go beyond the established motif finding methods based on large databases and sequence similarity, as described above, they will probably have to integrate structural and biophysical data along with large-scale sequencing results. At present, we cannot be sure how these algorithms could be designed. Artificial intelligence (AI) is currently revolutionizing many fields of computational biology, including the prediction of three-dimensional protein structures from linear sequences, an achievement considered highly improbable until very recently [30]. If ways can be found to muster the immense computer power required to do such calculations, AI may contribute to predict the consequences of SNVs on TF binding, gene regulation, and, ultimately, disease associations. This detailed understanding of the impact of genomic variants on patient phenotypes will increase the molecular diagnosis rate for patients with suspected rare genetic disease, leading to improved medical management for patients and their families.
